# Two Cases of Primary Aortoenteric Fistulas Diagnosed by Computed Tomography

**DOI:** 10.7759/cureus.63406

**Published:** 2024-06-28

**Authors:** Arianna Gregg, Morgan Sly, Todd Williams

**Affiliations:** 1 Department of Medical Education, University of Nevada, Reno School of Medicine, Reno, USA; 2 Department of Radiology, Henry Ford Health System, Detroit, USA

**Keywords:** primary aortoenteric fistula, acute gi bleed, diagnostic challenge, ct, primary aortaduodenal fistula

## Abstract

A primary aortoenteric fistula is a rare clinical entity that leads to severe upper gastrointestinal bleeding and carries a high risk of mortality, yet diagnosing aortoenteric fistulas remains challenging. Diagnosis is frequently delayed due to the uncommon and non-specific nature of the abdominal signs and symptoms. Rapid diagnosis and prompt surgical intervention are paramount to the successful management of this condition which is known for its profoundly poor prognosis. This report describes two cases of primary aortoenteric fistulas, one of which presented with melena and hematemesis, and the other presented with hematemesis and abdominal pain. In both cases, computed tomography angiography (CTA) demonstrated findings suggestive of an aortoenteric fistula, namely, locules of gas within the aortic lumen, which led to emergent surgical intervention. One patient underwent esophagogastroduodenoscopy while in the operating room before surgical intervention. One patient underwent repair with axillo-bifemoral bypass and the other with juxtarenal abdominal aortic aneurysm repair with a rifampin-soaked gelsoft dacron graft followed by primary bowel repair. Postoperative complications for one of the patients included duodenal repair breakdown as well as colonic ischemia. One patient made a meaningful recovery and remained without complications until the first postoperative visit two months after the repair. The other patient was discharged and then subsequently lost to follow-up. The two patients’ successful outcomes of such a lethal condition were in large part due to rapid diagnosis with CTA and prompt surgical intervention.

## Introduction

A primary aortoenteric fistula (PAEF) is a rare and life-threatening condition that occurs due to spontaneous communication between the aorta and intestinal tract [1]. This is in contrast to the more common secondary aortoenteric fistula (AEF) which forms following aortic interventions [1]. More specifically, a PAEF occurs when the native aneurysmal aortic wall erodes into the adjacent gastrointestinal tract (GI) tract, frequently leading to massive GI bleeding and rapid patient decompensation. The most common predisposing factors are atherosclerosis (60-85%) and infection (15%) [2]. A literature review of 366 cases revealed that 72.9% of PAEFs were in the duodenum, with 66% occurring in the third portion of the duodenum (D3) and 33% in the fourth portion (D4) [3]. These segments are at particular risk of fistula formation with the aorta as they are caught between the ligament of Treitz, the aorta, and the superior mesenteric artery, resulting in friction between the aorta and bowel with vessel pulsations and peristaltic waves [4]. Presentation varies but most commonly involves an upper GI bleed, along with a history of a prior aortic operation or the presence of an aortic aneurysm [5]. Patients can deteriorate rapidly, making swift diagnosis and early repair essential. Treatment options include open or endovascular repair, with the latter offering a less physiologically demanding approach that can serve as a bridge to definitive surgical management [6]. Despite these interventions, complications are common, and the mortality rate remains high for patients who develop AEFs with a near 100% mortality rate if left untreated and an approximately 50% mortality rate within 60 days after repair [6].

The rarity, non-specific symptoms, and unpredictable clinical course of AEF contribute to diagnostic and therapeutic complexities for treatment teams. Clinical suspicion and appropriate diagnostic evaluation are crucial to achieving timely intervention, which ultimately decreases the chances of mortality. This report describes two patients with GI bleeding whose contrast-enhanced computed tomography (CT) imaging raised suspicions for AEF, prompting emergent and successful surgical repair.

## Case presentation

Case 1

**Figure 1:**
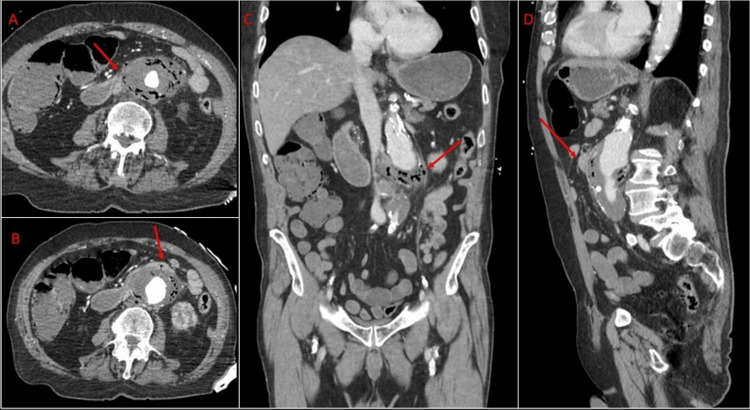
Computed tomography images of Case 1. A: Axial view demonstrating air within the aneurysm sac abutting the third and fourth portions of the duodenum (crossing the aneurysm anteriorly – arrow). B: Axial view shows the fistula tract evidenced by a focus of air extending through the duodenal wall and into the aneurysm sac (arrow). C: Coronal view showing the 6.5-cm infrarenal abdominal aortic aneurysm with air in the aneurysm sac (arrow). D: Sagittal view showing the aneurysm sac abutting the third portion of the duodenum anteriorly (arrow).

An 80-year-old male with a past medical history significant for a known abdominal aortic aneurysm (AAA), coronary artery disease, prior percutaneous coronary intervention, peripheral vascular disease, hypertension, dyslipidemia, and left subclavian stenosis presented to the hospital after being found down at home with blood around his mouth. He reported a history of melena for several days and one episode of coffee ground emesis before presentation. He also endorsed weakness, abdominal pain, and bilateral leg pain. Laboratory investigations on presentation were significant for a hemoglobin of 6.0 g/dL, lactate of 12 mg/dL, and a positive fecal occult blood test. Vital signs were significant for hypothermia to 33.9°C and tachypnea. CT angiography (CTA) demonstrated aneurysmal dilatation of the infrarenal abdominal aorta with irregular non-calcified intraluminal thrombus measuring up to 5.6 × 6.5 cm. There was notable irregular high-attenuation fluid extending along the margins of the aneurysm with intramural locules of gas, irregularity of the aortic wall, and local mass effect on an anterior loop of the proximal small bowel with a questionable fistulous tract (Figure 1). Together, these findings were concerning for contained rupture with air in the sac abutting the duodenum suggestive of an AEF. He was taken to the operating room (OR) emergently where esophagogastroduodenoscopy (EGD) showed a pulsating mass abutting the wall of the duodenum with erythematous mucosa but no active bleeding or luminal blood. Subsequent laparotomy confirmed a primary fistula with a defect in the duodenojejunal flexure at the level of the ligament of Treitz. A small bowel enterotomy, axillo-bifemoral bypass using an 8 mm Gore PTFE prefabricated graft, ligation of the infrarenal aorta, debridement of the aneurysmal sac, and creation of an omental flap which was placed between the graft and bowel. At the conclusion of the case, distal lower extremity Doppler signals were present. Cultures of the aortic thrombus grew *Streptococcus*, *Lactobacillus*, and *Veillonella* and Fungitell was positive. He was treated with six weeks of ampicillin, sulbactam, and fluconazole and transitioned to amoxicillin and clavulanic acid for indefinite suppressive therapy due to graft placement. He made a meaningful recovery and remained without complications until the first postoperative visit two months after the repair.

Case 2

**Figure 2:**
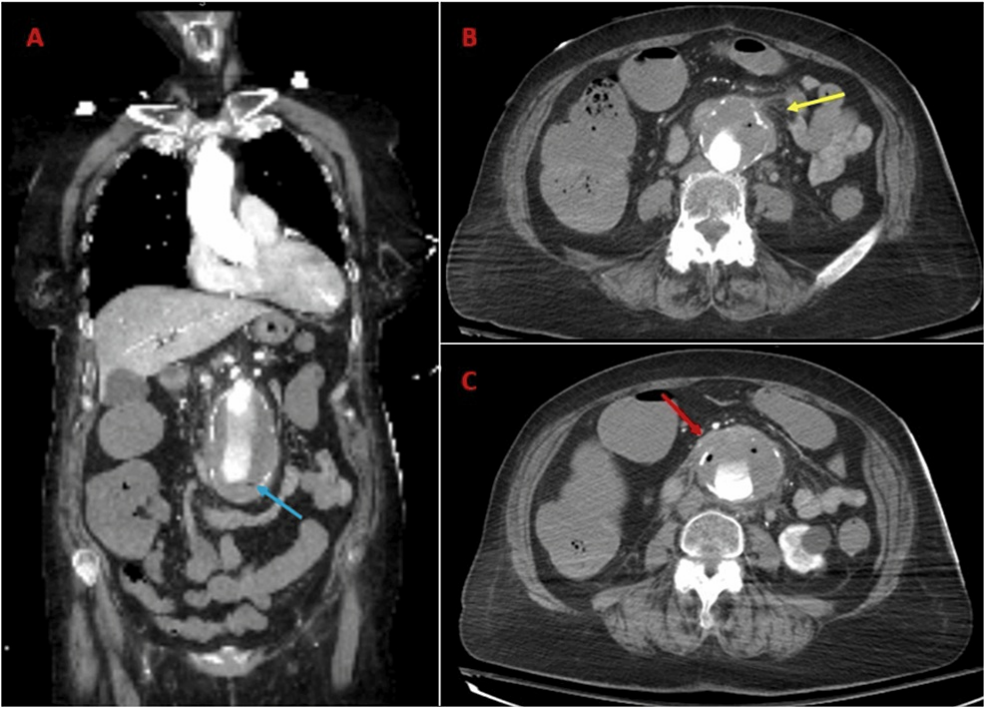
Computed tomography angiography images of Case 2. A and B: Coronal and axial views showing a 5.5-cm abdominal aortic aneurysm with extensive peripheral wall thrombus with a few foci of gas (blue arrow), aneurysm wall thickening, and peri-aortic inflammatory fat stranding (yellow arrow). C: Axial view showing the third portion of the duodenum (red arrow) abutting the aneurysm sac anteriorly.

A 79-year-old female with a past medical history of hypertension, chronic obstructive pulmonary disease, and tobacco use presented with abdominal pain and hematemesis. Laboratory results were significant for white blood cells of 16 × 10^9^/L, hemoglobin of 6.6 g/dL, and lactate of 14 mg/dL. The patient was subsequently transferred to the hospital for a higher level of care for a presumed AEF based on imaging and arrived intubated/sedated on fentanyl infusion. She became hypotensive requiring initiation of vasopressors and from the resuscitation bay was taken directly for an urgent CTA. CTA was performed and demonstrated a 5.5-cm infrarenal AAA with extensive wall thrombus with a few foci of gas, wall thickening, perianeurysmal inflammatory fat stranding, and mass effect on segment 3 of the duodenum concerning for a primary aorto-duodenal fistula (Figure 2). The patient was emergently taken to the OR and underwent juxtarenal AAA repair with a rifampin-soaked gelsoft dacron graft (22 mm tube), wide excisional debridement of infected retroperitoneum, primary repair of two D3 defects (one 3 mm long and the other 5 mm long), and the creation of a retro-colic pedicled omental flap that was positioned between the aortic graft and duodenum. Distal lower extremity Doppler signals were present at case completion. Her hospital course was complicated by a breakdown of both duodenal repairs requiring a return to the OR for repeat primary repair, as well as intermittent melena while on anticoagulation and acute tubular necrosis. Colonoscopy showed ischemic colitis of the descending and sigmoid colon which was managed supportively and she achieved full renal recovery without renal replacement therapy. OR cultures from the infected thrombus grew *Klebsiella pneumoniae*, *Candida albicans*, and *Streptococcus mitis* which were treated with six weeks of ceftriaxone and flagyl and 12 weeks of anidulafungin. The patient was subsequently lost to follow-up after discharge.

## Discussion

PAEFs are extremely rare, with an incidence rate of 0.07% [7]. Their clinical presentation varies widely, from general malaise to acute exsanguination [5]. Rarely, patients may display the classic triad of symptoms, namely, abdominal pain (32%), GI bleeding (64%), and a pulsating abdominal mass (25%) [8]. Around 70% of patients present with GI bleeding, known as a “herald bleed,” which resolves spontaneously due to vasospasm and clot formation, often followed by massive hemorrhage within hours to months [4,5]. The clinical progression can be very rapid, preventing adequate diagnostic tests such as endoscopy or CT from determining the cause of the GI bleeding [9].

As a PEAF is rare, clinical suspicion is often low. However, it should be considered in patients with a history of aortic aneurysms who present with GI bleeding, abdominal pain, or hemorrhagic shock. Diagnosing PAEF is challenging; only 36% of cases are diagnosed preoperatively [10]. EGD is often the first diagnostic test performed. However, the absence of active bleeding on endoscopy does not rule out an AEF, and the detection rate of endoscopy is only 25% [4]. Furthermore, endoscopic evaluation of the GI tract distal to D3, a common location of AEF, can be challenging due to the acute angulation between D3 and D4 [11].

The wide accessibility of CT machines in emergency departments and their non-invasive nature make CT an enticing diagnostic tool that can uncover findings suggestive of an AEF and provide surgeons with patient-specific anatomical information for surgical planning. Contrast-enhanced CT is the preferred imaging tool for diagnosis as it can reveal pathognomonic signs such as the extravasation of aortic contrast into the enteric lumen or foci of air within the aortic wall, the latter of which was seen in both of our cases [4]. Additional suggestive CT findings include contrast in the GI tract, irregularity within the surrounding aortic fat, disappearance of the fat plane between the aorta and adjacent viscera, hematoma within the retroperitoneum, bowel wall or lumen, and bowel wall thickening adjacent to the aneurysm [4,12]. In the presence of these additional findings, CTA has a sensitivity of 94% and a specificity of 85% in diagnosing AEF [5]. To ensure accurate diagnosis, radiologists should be familiar with pathologies that mimic AEFs radiologically [12]. These pathologies include retroperitoneal fibrosis, mycotic aortic aneurysms, and infectious aortitis [12]. However, retroperitoneal fibrosis typically involves the aorta, inferior vena cava, and ureters rather than the GI tract and does not demonstrate periaortic foci of gas. Infectious aortitis involves a normal caliber aorta with aortic wall thickening and intramural gas. Lastly, mycotic aortic aneurysms typically demonstrate brisk aneurysmal growth in the clinical setting of sepsis [12]. To achieve a proper diagnosis, radiologists must be aware of the characteristic imaging findings of AEF as well as those of the aforementioned mimicking conditions.

At our institution, CT evaluation includes a portal venous phase at 70 seconds from either above the lung apices or above the diaphragm to below the pubic symphysis, with 1.25-mm collimation. Images are reformatted to create sagittal and coronal images with a slice thickness of 2.5 mm. CTA evaluation of the native aorta includes an arterial phase with 1.25 collimation and reformatted axial, coronal, and sagittal images with a slice thickness of 2.5 mm and an axial maximum intensity projection. Oral contrast is not used as it can obscure arterial contrast filling the GI lumen.

In untreated cases of AEF, the mortality rate is almost certain, with surgical intervention being the only definitive treatment [6]. The primary goals of intervention are to control bleeding, manage infection, and maintain distal perfusion. Treatment options include endovascular aortic repair versus open repair, with the latter being associated with a lower rate of sepsis and ongoing infection postoperatively as open repair allows for infected tissue debridement and primary repair of the enteric tract [6]. Successful endovascular treatment of PAEF has been reported as an option for unstable patients who are unsuitable for open surgery and is reported to have a much lower in-hospital mortality rate (7%) when compared to open repair (34%) [13,14]. Interestingly, between open and endovascular repair there was no reported difference in AEF-free survival [6].

## Conclusions

We present two cases of GI bleeding where contrast-enhanced CT scans suggested AEFs, resulting in urgent and successful surgical repair. While positive surgical outcomes were seen in our two patients, identifying PAEFs is challenging, as diagnosis is often delayed due to the non-specific nature of the abdominal signs and symptoms, limited knowledge of AEF frequency, and appropriate use of diagnostic imaging. Although uncommon, an AEF must be considered as a potential cause of occult, intermittent, or massive upper GI bleeding in elderly patients with a known AAA presenting with abdominal pain due to its significant mortality and morbidity associated with delayed diagnosis. Several non-invasive and invasive imaging techniques exist to identify the source of upper GI bleeding. While no single diagnostic test is typically conclusive for an AEF, contrast-enhanced CTA can synergistically confirm or rule out a PAEF diagnosis swiftly and without jeopardizing patient safety. The present cases highlight the important role of clinical suspicion as well as urgent CT in achieving a quick diagnosis, prompt surgical repair, and, ultimately, an improved chance of patient survival.
